# Optimising sampling and analysis protocols in environmental DNA studies

**DOI:** 10.1038/s41598-021-91166-7

**Published:** 2021-06-02

**Authors:** Andrew Buxton, Eleni Matechou, Jim Griffin, Alex Diana, Richard A. Griffiths

**Affiliations:** 1 Durrell Institute of Conservation and Ecology, School of Anthropology and Conservation, University of Kent, Marlowe Building, Canterbury, Kent, CT2 7NR UK; 2 School of Mathematics, Statistics and Actuarial Science, University of Kent, Sibson Building, Canterbury, Kent, CT2 7FS UK; 3 Department of Statistical Science, University College London, 196-199 Tottenham Court Rd, Bloomsbury, London, W1T 7PJ UK

**Keywords:** Ecological modelling, Freshwater ecology, Conservation biology

## Abstract

Ecological surveys risk incurring false negative and false positive detections of the target species. With indirect survey methods, such as environmental DNA, such error can occur at two stages: sample collection and laboratory analysis. Here we analyse a large qPCR based eDNA data set using two occupancy models, one of which accounts for false positive error by Griffin et al*.* (J R Stat Soc Ser C Appl Stat 69: 377–392, 2020), and a second that assumes no false positive error by Stratton et al*.* (Methods Ecol Evol 11: 1113–1120, 2020). Additionally, we apply the Griffin et al*.* (2020) model to simulated data to determine optimal levels of replication at both sampling stages. The Stratton et al*.* (2020) model, which assumes no false positive results, consistently overestimated both overall and individual site occupancy compared to both the Griffin et al*.* (2020) model and to previous estimates of pond occupancy for the target species. The inclusion of replication at both stages of eDNA analysis (sample collection and in the laboratory) reduces both bias and credible interval width in estimates of both occupancy and detectability. Even the collection of > 1 sample from a site can improve parameter estimates more than having a high number of replicates only within the laboratory analysis.

## Introduction

Biodiversity assessments are increasingly carried out by indirect survey methodologies, such as the targeting of environmental DNA (eDNA). Such surveys rely on the collection, isolation and identification of DNA released from source organisms into the environment, to assess recent occupancy of a site by a species^1,2^. Both single-species qPCR and multi-species metabarcoding^3,4^ approaches to eDNA analysis infer that the detection of DNA indicates the presence of the target species. However, unlike direct observations of the species in the field, eDNA analysis involves two stages of sampling and analysis (i.e. field sampling and laboratory analysis) with imperfect detection possible at both stages^5^.

Optimising survey design to account for and reduce imperfect detection should be a key consideration in any study using eDNA^6,7^. Consideration needs to be given both to sample collection and laboratory analysis design, as well as downstream data processing. The level of error and therefore detectability, in a study targeting eDNA, will be influenced by several factors, relating to the species^8^, survey timing ^9^, concentration of DNA within the waterbody^9^, sample volume^10^, strategy used to collect a sample^11^, protocols used for DNA extraction^12^ and DNA amplification and identification protocols^4^. Taking these into consideration and refining the methodologies used will reduce error, but it would be naïve to assume this would be sufficient to ensure it was zero in all cases^7^. The number of sites, number of replicate samples collected from each site and level of replication undertaken within the laboratory^13,14^ will influence overall detectability. However, the use of statistical software and modelling approaches can also be considered to infer and account for site occupancy and the probabilities of observation error rates across a data set^7,15–18^ . As stated by Tingley et al*.*^19^ “Future eDNA studies should still estimate the likelihood of, and properly account for, false positive detections using modelling approaches”.

The levels of replication needed at different stages of the workflow have not been standardised across eDNA studies, with some undertaking replication at both the sample collection and analysis stages^20,21^, while others replicate only at the analysis stage^22^. The level of field replication is usually low, with a single sample often collected, or multiple samples collected and then combined for analysis, although up to ten samples per site have been collected and analysed independently^23^. At the laboratory stage, replication can vary considerably from three^24^, to 12^22^ in some quantitiative PCR (qPCR) base studies. The disparity between the levels of replication seen at stage 1 and stage 2 is likely to be due to replication at stage 1 being more logistically onerous than stage 2. The optimal levels of replication at field and laboratory stages have yet to be assessed in eDNA surveys. This is important, as insufficient levels of replication may lead to low confidence in results, while over-replication risks wasting resources that could be directed elsewhere.

**Figure 1:**
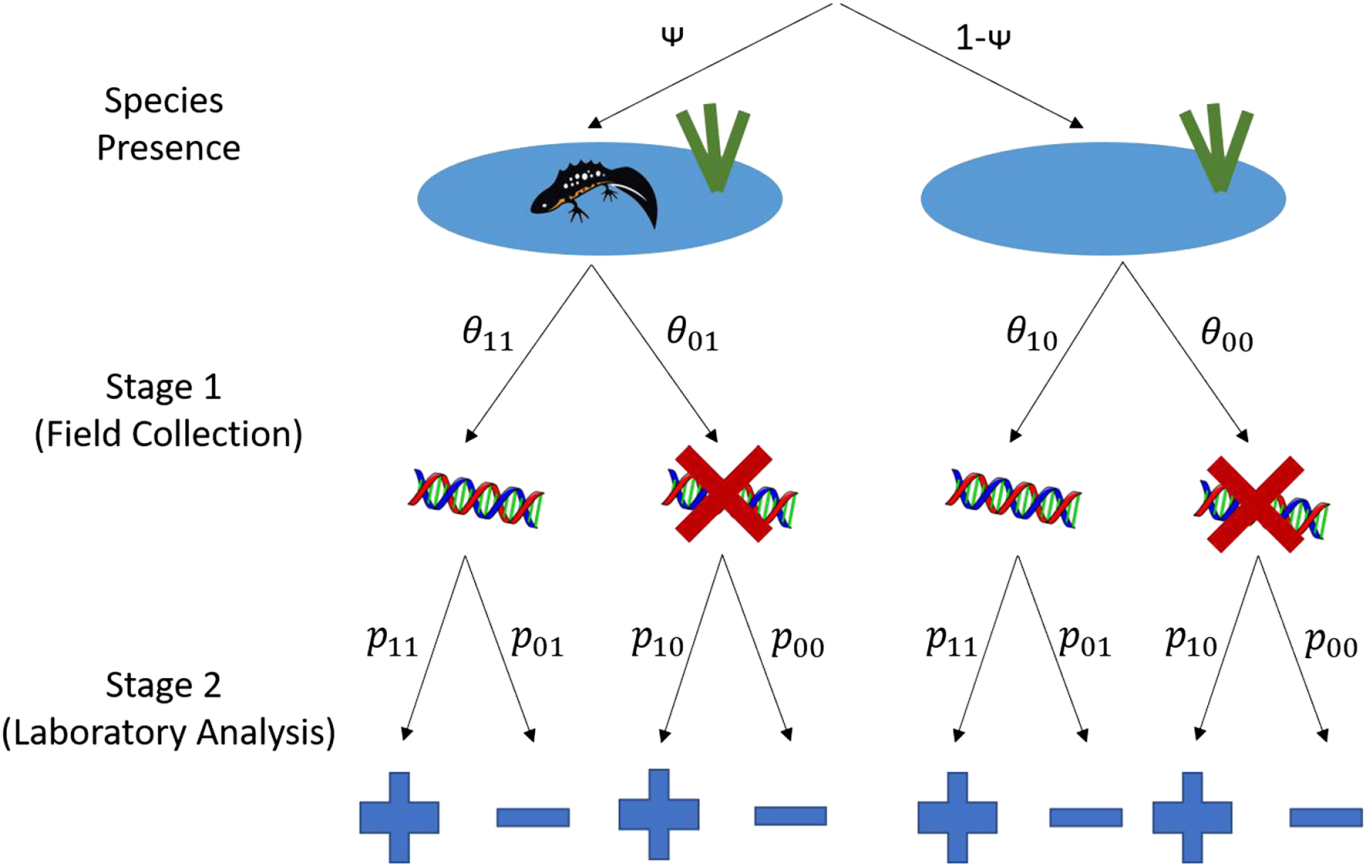
A schematic representation of the Griffin^16^ model demonstrating true and false results at both stages. ψ: species presence; ${\theta }_{11}$: Stage 1 true positive; ${\theta }_{01}$: Stage 1 false negative; ${\theta }_{00}$: stage 1 true negative; ${\theta }_{10}$: stage 1 false positive; ${p}_{11}$:Stage 2 true positive; ${p}_{01}$: Stage 2 false negative; ${p}_{00}$: Stage 2 true negative; ${p}_{10}$: Stage 2 false positive.

Validation of the eDNA survey method has usually been against other survey methods, using direct or indirect observations^25,26^. However, all survey methods suffer from imperfect detection: the target organism—or its DNA—may be present but not be detected (false negative error), or the target may be detected when it is in fact absent, a misidentification (false positive error)^5,27^. With surveys relying on direct observation of a species, there is only one stage where the error may occur. On the other hand, in eDNA surveys errors may occur at two levels—sample collection in the field (Stage 1) and sample analysis in the laboratory (Stage 2)^7^. Figure 1 shows a schematic representation of true and false results at both stages. The probabilities of false positive and negative errors and the probability of site occupancy may also be influenced by a variety of covariates that relate to environmental conditions, which could act at either Stage 1 or Stage 2^5^.

Occupancy models^28–30^ have been used extensively to model data collected by direct observation of the target species. More recently, multi-scale occupancy models, which account for the two stages of eDNA surveys, have also been proposed and implemented in R. Two examples are the EDNAOCCUPANCY R package^31^ and the msocc package^18^, with the latter implementing more efficient inference than the former, in a Bayesian framework using a Markov chain Monte Carlo (MCMC) method of sampling from a probability distribution. The msocc package by Stratton et al*.*^18^ also provides a web-based application to allow analysis via a user-friendly interface. These conventional occupancy models only account for the probability of a false negative observation error at the two eDNA survey stages. However, it is known that false positive errors are non-negligible in eDNA surveys^5,7,19^. For example, contamination of samples, misclassification of DNA sequences or poor specificity within the PCR process may all result in false positive detections. Guillera-Arroita et al*.*^32^ were the first to propose a model that accounts for both false negative and false positive observation error in the two stages of eDNA surveys. As is the case with similar models, (e.g. Royle and Link^27^) the model is only locally identifiable with eDNA data alone^33^. However, this limitation can be overcome via calibration from unambiguous survey methods or calibration experiments to identify core modelling parameters, both non-trivial exercises, or introducing informative prior distributions^19,32,34^. In addition, Guillera-Arroita et al*.*^32^ assumed that the probability of site occupancy as well as the probabilities of observation error are the same across all sites, an assumption that may not apply in all studies.

To circumvent these issues, Griffin et al*.*^16^ extended the Guillera-Arroita et al*.*^32^ model by considering a Bayesian approach and using informative prior distributions, to overcome identifiability issues and to allow for the estimation of occupancy and error at both field and laboratory stages to be functions of site covariates. The model can consider opportunistic confirmed presence–only data, but—unlike other modelling approaches—does not rely on it. We clarify here that, as is the case with conventional occupancy models, replication at the sample stage and analysis stage is necessary for all model parameters to be estimable. When there is no replication, for example when only a single sample has been collected from each site, then using opportunistic presence-only data, which are often easily obtainable, or modelling the probability of occupancy as a function of site covariates, helps overcome identifiability issues. These new models offer a unique opportunity to define levels of confidence in the results from eDNA data sets and to examine the effect of covariates on occupancy and detection at all stages of the analysis. An R shiny App has been created for the Griffin et al*.*^16^ model as presented in Diana et al*.*^34^, to provide a user-friendly application for the analysis of eDNA data for occupancy and error. The Diana et al*.*^34^ app implements an efficient MCMC algorithm for fitting the Griffin et al*.*^16^ model and for performing efficient Bayesian variable selection to identify important predictors of the probability of occupancy and the probabilities of observation error in both stages.

We apply the Griffin et al*.*^16^ model to simulated data to demonstrate its utility at different scales (number of sites) and the effect of replication at both sample collection and laboratory stages. We also apply it to a large eDNA data set targeting the great crested newt (*Triturus cristatus*) using the qPCR analysis method. We describe the performance of the model and the results it generates in terms of estimating the probabilities of site occupancy and of observation error at both stages and compare this to the output from the Stratton et al*.*^18^ model.

## Results

### Simulated data

The aim of the simulation was twofold: (1) to identify both optimum number of field samples (M) and qPCR replication (K) over a range of sample sizes (S); and (2) to identify the minimum number of sites required to provide reliable estimates of occupancy and error.

**Figure 2:**
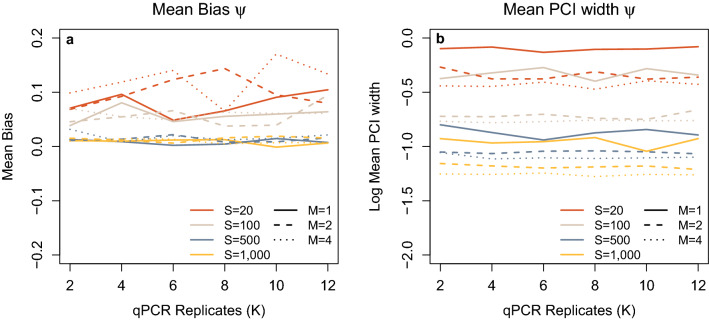
(**a**) Mean bias in occupancy (ψ) estimate with varying numbers of sites (S), samples collected (M) and qPCR replication (K). (**b**) Mean 95% PCI width for occupancy (ψ) plotted on a logarithmic scale, with varying numbers of sites (S), samples collected (M) and qPCR replication (K).

**Figure 3:**
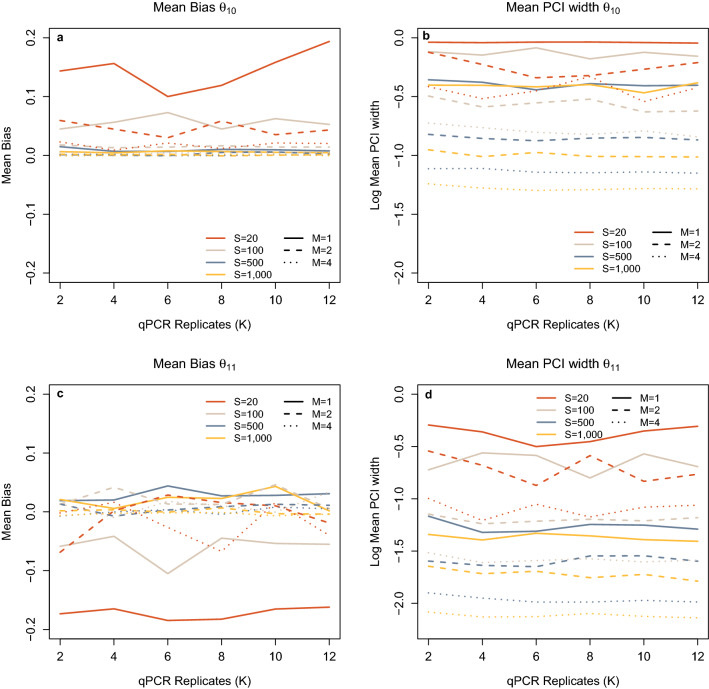
(**a**) Mean Bias in Stage 1 false positives (${\theta }_{10}$) estimate with varying numbers of sites (S), samples collected (M) and qPCR replication (K). (**b**) Mean 95% PCI width for Stage 1 false positives (${\theta }_{10}$) plotted on a logarithmic scale, with varying numbers of sites (S), samples collected (M) and qPCR replication (K). (**c**) Mean Bias in Stage 1 true positives (${\theta }_{11}$) estimate with varying numbers of sites (S), samples collected (M) and qPCR replication (K). (**d**) Mean 95% PCI width for Stage 1 true positives (${\theta }_{11}$) plotted on a logarithmic scale, with varying numbers of sites (S), samples collected (M) and qPCR replication (K).

**Figure 4:**
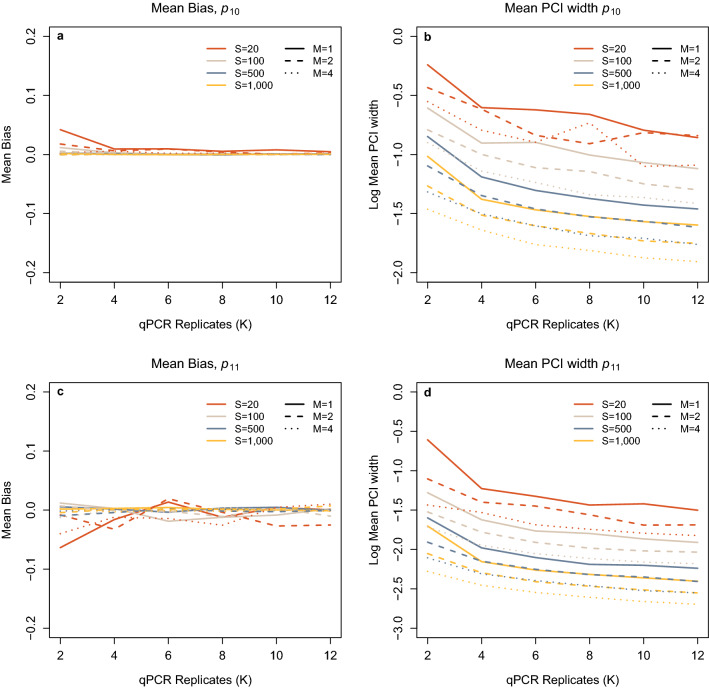
(**a**) Mean Bias in Stage 1 false positives (${p}_{10}$) estimate with varying numbers of sites (S), samples collected (M) and qPCR replication (K). (**b**) Mean 95% PCI width for Stage 1 false positives (${p}_{10}$) plotted on a logarithmic scale, with varying numbers of sites (S), samples collected (M) and qPCR replication (K). (**c**) Mean Bias in Stage 1 true positives (${p}_{11}$) estimate with varying numbers of sites (S), samples collected (M) and qPCR replication (K). (**d**) Mean 95% PCI width for Stage 1 true positives (${p}_{11}$) plotted on a logarithmic scale, with varying numbers of sites (S), samples collected (M) and qPCR replication (K).

M, S and K are all important in reducing the bias and mean posterior credible interval (PCI) widths for all or some of occupancy (ψ), stage 1 false positive ($${\theta }_{10})$$, stage 1 true positive ($${\theta }_{11})$$, stage 2 false positive ($${p}_{10})$$ and stage 2 true positive ($${p}_{11})$$ (Figs. 2, 3 and 4). For ψ there is some bias leading to an over-estimation of occupancy when S is low (either 20 or 100), but this becomes negligible when S increases to at least 500 (Fig. 2A). Neither M nor K influenced bias in ψ. The mean PCI width for ψ reduced between 21% (S = 500, K = 6) and 66% (M = 100, K = 10) when M was increased from one to two and to some extent depended on the individual combinations of K and S. However, when M was increased from two to four the reduction was between 1% (S = 500, K = 2) and 33% (S = 20, K = 2) again dependant on K and S. With increasing S, the mean PCI width of ψ also dropped sharply at all levels of M (Fig. 2B).

Increasing M and S led to a reduction in bias for both ${\theta }_{10}$ and ${\theta }_{11}$ (Stage 1 false and true positives) (Fig. 3A,C). Increasing M from one to two reduces ${\theta }_{10}$ bias at S = 20 and S = 100 considerably; however, increasing sampling further to M = 4 yields little increase in accuracy. When S was large (S > 500), increasing M also reduced bias; however, these were not as prominent (Fig. 3A,C). Again, for ${\theta }_{11}$ raising the number of samples from one to two increases accuracy substantially, an increase to M = 4 when S was low did not show any further reduction in bias. At higher levels of S, the reduction in mean bias for ${\theta }_{11}$ with increasing M was less pronounced. In a similar manner to ψ, a reduction of mean PCI width with an increase in M and S was seen within the data for both ${\theta }_{10}$ and ${\theta }_{11}$ (Fig. 3B,D), though the PCI width is wider for ${\theta }_{10}$ and ${\theta }_{11}$ than observed for the ψ estimate.

For both ${p}_{10}$ and ${p}_{11}$ mean bias is negligible when S ≥ 500, at all values for K and M. However, when S is low, we do see a reduction in bias with both increasing M and K (Fig. 4A,C). When S is low, the reduction in bias with increasing qPCR replicates is very strong between K = 2 and K = 4 with some improvement up to K = 6. However, above this level, increases in K have very little impact on Stage 2 bias. Increasing M from one to two—and to a lesser extent between two to four—does reduce ${p}_{10}$ bias, however its influence on ${p}_{11}$ was limited. As with mean bias, ${p}_{10}$ demonstrates an improvement in mean PCI width with an increase in all three M, S and K (Fig. 4B). The reduction in mean PCI width for ${p}_{10}$ with increased K is most pronounced between K = 2 and K = 6 and is observed at all levels of M and S, however when M = 1 an improvement is also seen up to the maximum level for K simulated (Fig. 4B). Posterior mean estimates with credible intervals for ψ, ${\theta }_{10}$, ${\theta }_{11}$, ${p}_{10}$ and ${p}_{11}$ can be found in Supplementary Figures S4, S5, S6, S7, and S8.

### Evaluating model performance using great crested newt eDNA data

In the real great crested newt data set, 72% of the samples returned no positive qPCR replicates (Supplementary Fig. S1). Those that returned at least one positive qPCR replicate most commonly either returned all 12 replicates as positive (23%), or only one replicate as positive (17%), indicating a bimodal distribution in the frequency of qPCR replicates amplifying (Supplementary Fig. S2). Naïve occupancy can be set at different thresholds. If a single positive qPCR is used to define occupancy, then the naïve rate is 0.28. However, this reduces if two (0.23) or three (0.20) positive qPCR replicates are used as the threshold for defining occupancy. The presence of the species was confirmed by direct observation in only 53 of the 2958 sites, with 41% of these returning all 12 qPCR replicates as positive (Supplementary Fig. S3). However, 13% of surveys that revealed the presence of the species by direct observation yielded zero qPCR amplification (Figure S3), demonstrating confirmed false negative results.

**Figure 5:**
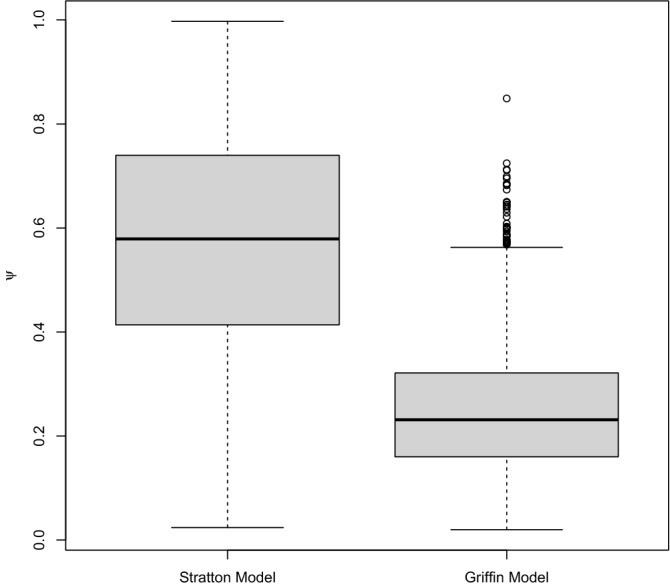
Boxplot of site posterior mean occupancy estimates for the Stratton et al*.*^18^ model (Left) and Griffin et al*.*^16^ model (Right). The median value for all sites, with inter-quartile range, 1.5 inter-quartile range inner fence and suspected outliers presented.

The posterior mean of the probability of pond occupancy at the baseline level was found to be 0.26 (PCI: 0.14–0.41) when assessed using the Griffin et al*.*^16^ model (Table 1), very close to the naïve estimate of 0.28 for occupancy assigned using a single positive qPCR replicate. However, the mean PCIs were wide and encompassed naïve estimates at various naïve occupancy selection thresholds. In contrast, the Stratton et al*.*^18^ model was found to have a higher posterior mean estimate for occupancy of 0.47 (PCI: 0.35–0.65; Fig. 5). Although there is some overlap of the credible intervals, the posterior means of the two models do not fall within the intervals for each other, demonstrating the risk of overestimating the probability of occupancy when false positive observation error is ignored. The probability of a true positive observation is estimated as lower for the Stratton et al*.*^18^ model at both Stage 1 and Stage 2 compared to the Griffin et al*.*^16^ model although credible intervals for true positive results at Stage 1 overlap (Table 1).

**Table 1:** Posterior mean estimates and 95% CI for occupancy, true and false positive error estimates using both the Stratton et al*.*^18^ model and Griffin et al*.*^16^ model.

	Stratton et al*.* Model^18^	Griffin et al*.*^16^ Model
Stage 1 True Positive	0.59 (PCI: 0.42–0.77)	0.77 (PCI: 0.57–0.92)
Stage 1 False Positive	0	0.02 (PCI: 0.003–0.08)
Stage 2 True Positive	0.55 (PCI: 0.54–0.56)	0.84 (PCI: 0.81–0.87)
Stage 2 False Positive	0	0.02 (PCI: 0.01–0.04)
Occupancy	0.47 (PCI: 0.35–0.65)	0.26 PCI: (0.14–0.41)

**Figure 6:**
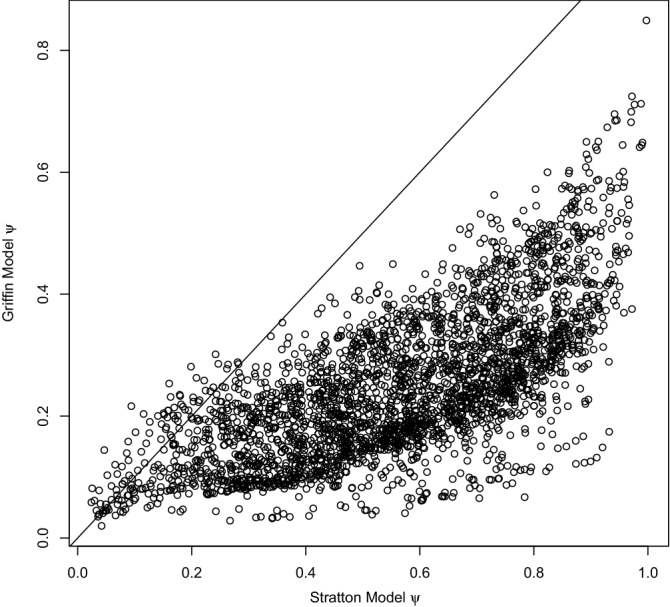
Paired site posterior mean occupancy probabilities for the Stratton et al*.*^18^ model (x-axis) and Griffin et al*.*^16^ model (y-axis), the black line represents the line through the origin.

Due to the inclusion of covariates, we can compare the site (pond) specific occupancy estimates for the two models. The Stratton et al*.*^18^ model was found to produce a higher site-specific occupancy estimate for 97.4% of sites (Fig. 6). Similarly, we examined the mean PCI width for site-specific occupancy estimates between the two apps, with the Stratton et al*.*^18^ model found to have a larger width at 95.1% of sites, with PCI only overlapping for 40.5% of sites. Without exception, for sites where there is no PCI overlap, the Stratton et al*.*^18^ model overestimated occupancy compared to the Griffin et al*.*^16^ model.

**Figure 7:**
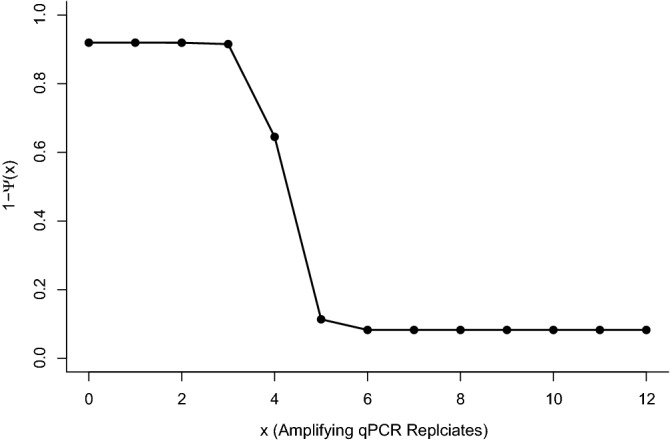
The posterior conditional probabilities of species absence (1 − ψ (x)), given x amplifying qPCR replicates, calculated using the Griffin et al*.*^16^ model.

The Griffin et al*.*^16^ model allows for the conditional probability of species absence to be calculated for x number of positive qPCR replicates for a baseline site, i.e. when all continuous covariates are equal to 0 and all categorical covariates are equal to their baseline level. With low numbers of replicates amplifying, the conditional probability of species absence is close to one and remains largely unchanged when fewer than three qPCR replicates amplify. The conditional probability of occupancy then changes rapidly between three and five replicates, before levelling off. There is little increase in occupancy estimates above six positive qPCR replicates, (Fig. 7).

## Discussion

Optimising survey design, and identifying the number of sites, samples, and laboratory replication, needs to be taken into consideration at the planning stage for any eDNA study. A compromise between replication, unnecessary effort and cost needs to be met. Using the Griffin et al*.*^16^ model, with the simulation input parameters for true occupancy (0.1), ${\theta }_{10}$ (0.01), ${\theta }_{11}$ (0.85), ${p}_{10}$ (0.01) and ${p}_{11}$ (0.9); we find that very little replication either at Stage 1 or Stage 2 can lead to biases and wide credible intervals in the occupancy and error estimates. This error is exacerbated when the number of sites is low (20 or 100 sites in our analysis). However, replication at Stage 1 seems to reduce bias across the three parameters, to a greater extent than replication at Stage 2; had K = 1 been included it is likely we would have also seen a large reduction in error between K = 1 and K = 2. With two samples collected from each site and analysed using between four and six qPCR replicates, less bias and narrower credible intervals are obtained than where only a single sample is collected but analysed with a high number of laboratory replicates. This is irrespective of number of sites visited. Increasing the number of qPCR replicates (K) does improve ${p}_{10}$ and ${p}_{11}$ bias and mean PCI width: this is expected as they operate at Stage 2, although the overall occupancy estimate was unaffected. Most of the improvement in ${p}_{10}$ and ${p}_{11}$ bias and mean PCI width is seen when Stage 2 replication is increased from two to six replicates, with minimal improvement above this level. Again, this is most prominent in studies with low numbers of sites.

Within eDNA studies, replication is usually focused at Stage 2, with no or limited replication at Stage 1. A widely used commercial example of this is the great crested newt commercial protocol within the UK^35^, where a single field sample is collected, extracted and then analysed using 12 qPCR replicates. A suitable compromise, which would minimise both error and effort (based on the input parameters for this simulation), would be two field samples per site each of which should be analysed four- to six-times using qPCR. It is assumed within this analysis that site occupancy does not change between field sampling events. i.e. multiple samples are collected at the same time. Our simulation study suggests that when the number of sites is low, some bias in the inference may be expected, depending on the level of replication in both stages. Our suggested level of replication (i.e. M = 2; K = 4–6) would be cost-effective compared to the current protocol for great crested newts (i.e. M = 1; K = 12), providing the same or a net reduction in the total number of qPCR runs, which is where most of the analytical costs lie.

The existence of false negative results is widely accepted^20,21^, and their existence was demonstrated in our data with 13% of samples where occupancy had been confirmed by direct observation failing to show any amplification. However, the bimodal distribution of amplification in terms of the number of qPCR replicates amplifying across all samples (Figs. S1 and S2), indicates that a low proportion of qPCR replicates amplifying may reflect false positives. Peaks where a high proportion of replicates amplify, are likely to be cases where the amount of eDNA in a sample is greater than a limiting threshold for amplification. Nonetheless, without considering false positive error, no similar justification exists for peaks in amplification with a low number of positive replicates, i.e. the number of samples amplifying 1/12 in a data set being considerably greater than the background level of amplification, where between 3/12 and 10/12 replicates amplify. Additionally, the posterior conditional probability of species absence is high when only a small proportion of qPCR replicates amplify. This supports false positives being more likely to be represented by samples where only a few replicates amplify, and that it is important to consider them in the analysis^5,19^. Interpretations about presence-absence using eDNA have so far relied on arbitrary thresholds about the number of positive qPCR replicates required to indicate presence. We have shown that naïve occupancy estimates for a threshold of one, two or three positive qPCR replicates, all fall within the modelled credible intervals for occupancy for our case study of great crested newts, using the Griffin et al*.*^16^ model. However, to minimise false positives a single amplifying qPCR replicate may not be the most appropriate threshold for assigning naïve occupancy to a site and a higher threshold should be applied.

The consequences of ignoring error rates when interpreting data are wide-ranging, and could have negative implications for conservation policy, implementation, and success. As a result, modelled occupancy and error rates need to be as unbiased and precise as possible. Here we examine the outputs from two models on the same data. The Griffin et al*.*^16^ and Stratton et al*.*^18^ models provide different estimates for occupancy for the same data, with the Griffin et al*.*^16^ model suggesting an overall occupancy rate of 0.26, while the Stratton et al*.*^18^ model was much higher at 0.47. Estimates for pond occupancy by great crested newts have been widely studied using conventional survey methodologies with rates for the core range reported as between 0.31 and 0.35^36^. However, naïve estimates of 0.13 may be more representative in marginal areas^37^. The great crested newt data used in this analysis were collected from across the range of the species in England. As such we would expect the posterior mean occupancy estimate to fall below that of the core range but above that of the marginal range, which is observed within the Griffin et al*.*^16^ model. However, the estimate returned by the Stratton et al*.*^18^ model is greater than previous occupancy estimates. A combination of failure to incorporate false positive error and the relatively low estimates for detectability observed in the Stratton et al*.*^18^ model (0.59 at Stage 1 and 0.55 at Stage 2) are the likely cause of the high occupancy estimate.

Within the Griffin et al*.*^16^ model, Stage 1 error demonstrated only a small degree of false positive results (0.02; PCI: 0.003–0.08). While the probability of a true positive was 0.77 (PCI: 0.52–0.92), this equates to a false negative rate of 23%. However, the PCI around these figures are wide, with the false negative rate potentially being as high as 48%. This is likely a result of the lack of replication at the sample collection phase; with the addition of Stage 1 replication, these PCIs would decrease in width and provide more informative estimates. With Stage 2 false positive error of 0.02 (PCI: 0.01–0.04), and Stage 2 true positive rate of 0.84 (PCI: 0.81–0.87), this equates to a false negative rate of 16%. PCI at Stage 2 were found to be narrower than in Stage 1, due to the higher level of replication. This results in an overall estimate for occupancy closer to the naïve estimate, and closer to most observed in nature, than the estimate observed with the Stratton et al*.*^18^ model.

The true site occupancy within the case study data set is unknown, as a result we cannot definitively present one of the two models examined here as closer to a true value than the other. However, we show using simulated data with similar input parameters (i.e., M = 1, S = 1000, K = 12) that the Griffin et al*.*^16^ model yields low bias and high precision. As a result, we can assume a high degree of confidence in the estimate given by the Griffin et al*.*^16^ model using these parameters, due to the large number of sites included in the case study data set. Based on the simulations we would recommend that sampling protocols include true replication at Stage 1 as well as retaining some replication at Stage 2. However, the level of Stage 2 replication that is seen in some protocols may be unnecessary.

## Methods

### Simulated data

Data was simulated using R version 4.0.2^38^ with estimates for occupancy (ψ), and with probabilities corresponding to observation in Stage 1 (${\theta }_{11}, {\theta }_{10}$) and Stage 2 (${p}_{11}, {p}_{10}$) , within levels we deemed to be representative of real-world conditions and broadly similar to those published in Griffin et al*.*^16^ and Diana et al*.*^34^*,* based on data collected for great crested newts. In all simulated data sets, occupancy (ψ) was set to 0.1, while ${\theta }_{11},$ or field sampling true positive rate set at 0.85, ${\theta }_{10}$ or field sampling false positive rate set to 0.01. ${p}_{11}$ or laboratory true positive rate at 0.9, with ${p}_{10}$ or laboratory false positive rate set at 0.01. Data sets were simulated varying *S*, the number of independent sites (20, 100, 500, 1000); *M,* the number of independent water samples (1, 2, 4); and *K,* the number of independent qPCR replicates (2, 4, 6, 8, 10, 12), with ten repeats of each possible combination of S, M and K. As explained in the introduction, when M or K are equal to 1 either confirmed presence data or covariates need to be considered to allow ψ and ${\theta }_{10}$ or ${\theta }_{11}$ and ${p}_{10}$ or ${p}_{11}$ to be distinguishable from one another^34^. To accommodate this, a continuous covariate centred at zero and a binary covariate with a probability of occurrence at 0.5, were included within the simulations.

Each data set was fitted using the Griffin et al*.*^16^ model with the default prior distributions, with MCMC runs set at 1 chain, 1000 burn-in iterations, 2000 iterations and thinning equal to 10. We calculated the mean difference between the true value used to simulate the data in each case and the posterior mean (referred to as bias), as well as the average width of 95% posterior credible intervals (PCI) across the 10 runs, for each combination of K, M and S and for each probability ψ, ${\theta }_{10}$, ${\theta }_{11}$, ${p}_{10}$ and ${p}_{11}$.

### Evaluating model performance using great crested newt eDNA data case study

We compared the performance of the Griffin et al*.*^16^ model and Stratton et al*.*^18^ model using a large eDNA data set collected as part of a broader programme to mitigate the impacts of development on great crested newts within the UK. eDNA samples were collected from a total of 2958 ponds (sites; S = 2958) across England in 2019, following the methodologies outlined in Biggs et al*.*^22^. In sum, twenty subsamples were collected from around the pond and pooled to give a single water sample per pond (M = 1). The samples were analysed in a commercial laboratory with 12 qPCR replicates (K = 12). If any life stage of the species was observed directly while taking the eDNA sample this was noted, but no targeted observational surveys for newts were undertaken. In addition, ten covariates that relate to the suitability of the pond and its immediate surrounding were collected at all survey sites using the method described by Oldham et al*.*^39^.

All continuous covariates were manually standardised prior to the analysis to have mean 0 and standard deviation 1, and sites with inconclusive eDNA results (signs of degradation or PCR inhibition) were removed. The Diana et al*.*^34^ app was run locally in R 4.0.2^38^. The number of burn-in iterations and iterations in one chain were both kept at the default settings of 3000. The prior distributions were unchanged from the default settings. Probabilities for occupancy, Stage 1 and Stage 2 true and false positive results were considered as functions of all available covariates.

The Stratton model was run locally in R 4.0.2^38^. This app requires qPCR replicates to be individually assigned, whereas the data used was only made available as the total number of positive replicates. As a result, we randomly assigned the appropriate number of positive and negative qPCR replicates so that each sample had the correct number. The app was run as outlined in Stratton et al*.*^18^ using a model where site was a function of covariates, but sample and laboratory replication were not. The default 1000 MCMC samples was used.

## Supplementary Information

Supplementary Information.

## Data Availability

The raw data used in the model comparison case study was collected by Natural England and is available through the Natural England Open Data Portal https://naturalengland-defra.opendata.arcgis.com/datasets/ffba3805a4d9439c95351ef7f26ab33c_0/data. The simulated data output and simulation code has been uploaded to Kent Academic Repository (https://kar.kent.ac.uk/88282/).

## References

[1] Jane SF (2015). Distance, flow and PCR inhibition: eDNA dynamics in two headwater streams. Mol. Ecol. Resour..

[2] Thomsen PF, Willerslev E (2015). Environmental DNA: An emerging tool in conservation for monitoring past and present biodiversity. Biol. Conserv..

[3] Valentini A (2016). Next-generation monitoring of aquatic biodiversity using environmental DNA metabarcoding. Mol. Ecol..

[4] Harper LR (2018). Needle in a haystack? A comparison of eDNA metabarcoding and targeted qPCR for detection of great crested newt (*Triturus cristatus*). Ecol. Evol..

[5] Ficetola GF (2015). Replication levels, false presences and the estimation of the presence/absence from eDNA metabarcoding data. Mol. Ecol. Resour..

[6] Willoughby JR, Wijayawardena BK, Sundaram M, Swihart RK, DeWoody JA (2016). The importance of including imperfect detection models in eDNA experimental design. Mol. Ecol. Resour..

[7] Burian A (2021). Improving the reliability of eDNA data interpretation. Mol. Ecol. Resour..

[8] Klymus KE, Richter CA, Chapman DC, Paukert C (2015). Quantification of eDNA shedding rates from invasive bighead carp *Hypophthalmichthys nobilis* and silver carp *Hypophthalmichthys molitrix*. Biol. Conserv..

[9] Buxton AS, Groombridge JJ, Zakaria NB, Griffiths RA (2017). Seasonal variation in environmental DNA in relation to population size and environmental factors. Sci. Rep..

[10] Mächler E, Deiner K, Spahn F, Altermatt F (2016). Fishing in the water: Effect of sampled water volume on environmental DNA-based detection of macroinvertebrates. Environ. Sci. Technol..

[11] Spens J (2016). Comparison of capture and storage methods for aqueous macrobial eDNA using an optimized extraction protocol: Advantage of enclosed filter. Methods Ecol. Evol..

[12] Djurhuus A (2017). Evaluation of filtration and DNA extraction methods for environmental DNA biodiversity assessments across multiple trophic levels. Front. Mar. Sci..

[13] Lugg, W. H., Griffiths, J., van Rooyen, A. R., Weeks, A. R. & Tingley, R. Optimal survey designs for environmental DNA sampling. *Methods Ecol. Evol.* **9**, 1049–1059 (2017).

[14] Mauvisseau Q (2019). Influence of accuracy, repeatability and detection probability in the reliability of species-specific eDNA based approaches. Sci. Rep..

[15] Willoughby, J. R., Wijayawardena, B. K., Sundaram, M., Swihart, R. K. & DeWoody, J. A. The importance of including imperfect detection models in eDNA experimental design. *Mol. Ecol. Resour.* **16 **, 837–844 (2016). 10.1111/1755-0998.12531 27037675

[16] Griffin JE, Matechou E, Buxton AS, Bormpoudakis D, Griffiths RA (2020). Modelling environmental DNA data; Bayesian variable selection accounting for false positive and false negative errors. J. R. Stat. Soc. Ser. C Appl. Stat..

[17] Lahoz-Monfort JJ, Guillera-Arroita G, Tingley R (2016). Statistical approaches to account for false-positive errors in environmental DNA samples. Mol. Ecol. Resour..

[18] Stratton C, Sepulveda AJ, Hoegh A (2020). msocc: Fit and analyse computationally efficient multi-scale occupancy models in r. Methods Ecol. Evol..

[19] Tingley R, Coleman R, Gecse N, van Rooyen A, Weeks A (2020). Accounting for false positive detections in occupancy studies based on environmental DNA: A case study of a threatened freshwater fish (*Galaxiella pusilla*). Environ. DNA.

[20] Schmidt BR, Kéry M, Ursenbacher S, Hyman OJ, Collins JP (2013). Site occupancy models in the analysis of environmental DNA presence/absence surveys: A case study of an emerging amphibian pathogen. Methods Ecol. Evol..

[21] Vörös J, Márton O, Schmidt BR, Gál JT, Jelić D (2017). Surveying Europe’s only cave-dwelling chordate species (*Proteus anguinus*) using environmental DNA. PLoS ONE.

[22] Biggs J (2015). Using eDNA to develop a national citizen science-based monitoring programme for the great crested newt (*Triturus cristatus*). Biol. Conserv..

[23] Cantera I (2019). Optimizing environmental DNA sampling effort for fish inventories in tropical streams and rivers. Sci. Rep..

[24] Dejean T (2012). Improved detection of an alien invasive species through environmental DNA barcoding: The example of the American bullfrog *Lithobates catesbeianus*. J. Appl. Ecol..

[25] Eiler A, Löfgren A, Hjerne O, Nordén S, Saetre P (2018). Environmental DNA (eDNA) detects the pool frog (*Pelophylax lessonae*) at times when traditional monitoring methods are insensitive. Sci. Rep..

[26] Nakagawa H (2018). Comparing local- and regional-scale estimations of the diversity of stream fish using eDNA metabarcoding and conventional observation methods. Freshw. Biol..

[27] Royle JA, Link WA (2006). Generalized site occupancy models allowing for false positives and false negative errors. Ecology.

[28] Mackenzie DI, Kendall WL (2002). How should detection probability be incorporated into estimates of relative abundance?. Ecology.

[29] MacKenzie DD, Nichols JD, Hines JE, Knutson MG, Franklin AB (2003). Estimating site occupancy, colonization, and local extinction when a species is detected imperfectly. Ecology.

[30] Tyre AJ, Tenhumberg B, Field SA, Niejalke D, Possingham HP (2003). Improving precision and reducing bias in biological surveys: Estimating false-negative error rates. Ecol. Appl..

[31] Dorazio RM, Erickson RA (2018). EDNAOCCUPUANCY: An R package for multi-scale occupancy modeling of environmental DNA data. Mol. Ecol. Resour..

[32] Guillera-Arroita G, Lahoz-Monfort JJ, van Rooyen AR, Weeks AR, Tingley R (2017). Dealing with false positive and false negative errors about species occurrence at multiple levels. Methods Ecol. Evol..

[33] Cole DJ (2020). Parameter Redundancy and Identi Ability.

[34] Diana A, Matechou E, Griffin JE, Buxtron AS, Griffiths RA (2020). An Rshiny app for modelling environmental DNA data: Accounting for false positve and false negative observation error. bioRxiv.

[35] Biggs, J. *et al. Analytical and methodological development for improved surveillance of the great crested newt. Defra Project WC1067.* (2014).

[36] Sewell D, Beebee TJC, Griffiths RA (2010). Optimising biodiversity assessments by volunteers: The application of occupancy modelling to large-scale amphibian surveys. Biol. Conserv..

[37] Buxton AS, Tracey H, Downs NC (2021). How reliable is the habitat suitability index as a predictor of great crested newt presence or absence?. Herpertological J..

[38] R-Core Team. R: language and environment for statistical computing. (2020).

[39] Oldham RS, Keeble J, Swan MJS, Jeffcote M (2000). Evaluating the suitability of habitat for the great crested newt (*Triturus cristatus*). Herpetol. J..

