# Supplementary material for: Optimising sampling and analysis protocols in environmental DNA studies
Source: Sci Rep. 2021 Jun 2;11:11637. doi: 10.1038/s41598-021-91166-7 (PMC8172848; doi:10.1038/s41598-021-91166-7)
Supplement: Supplementary file 1 — Supplementary Information. [file 41598_2021_91166_MOESM1_ESM.pdf]

# Optimising sampling and analysis protocols in environmental DNA studies – Supplementary Information

Buxton A.S., Matechou E., Griffin J., Diana A., and Griffiths R.A.

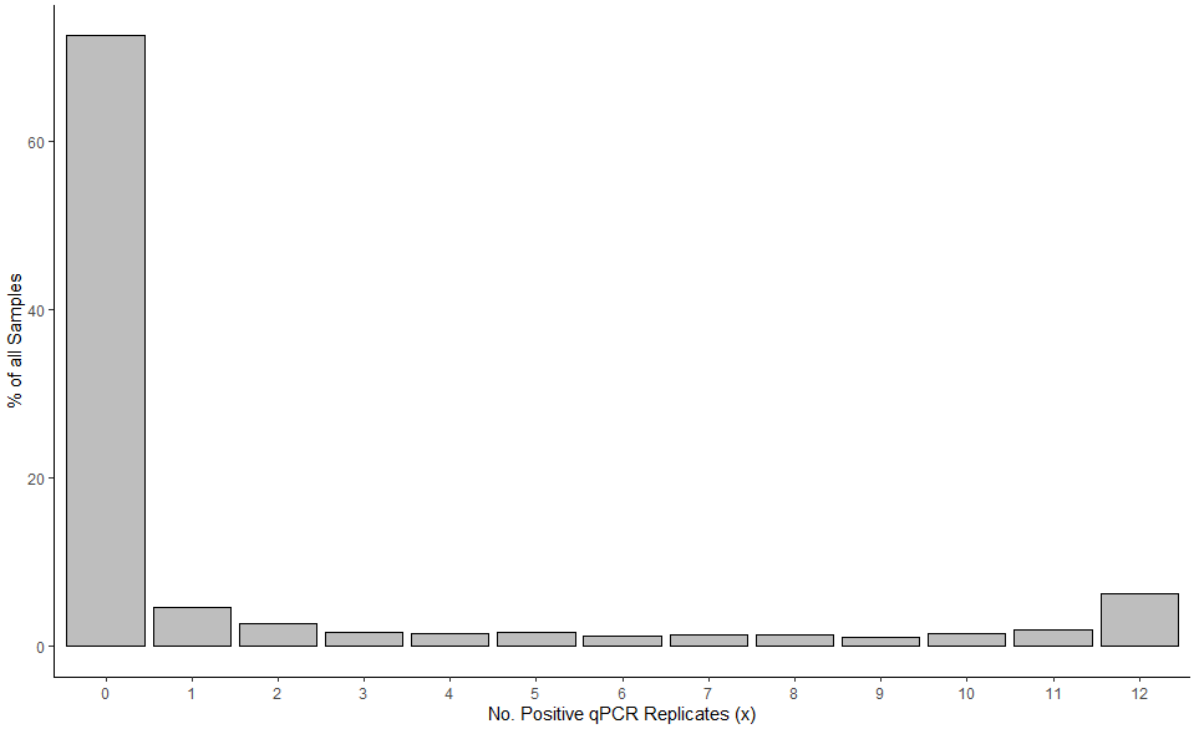


Figure S1 - Percentage of all samples with x number of amplifying qPCR replicates.

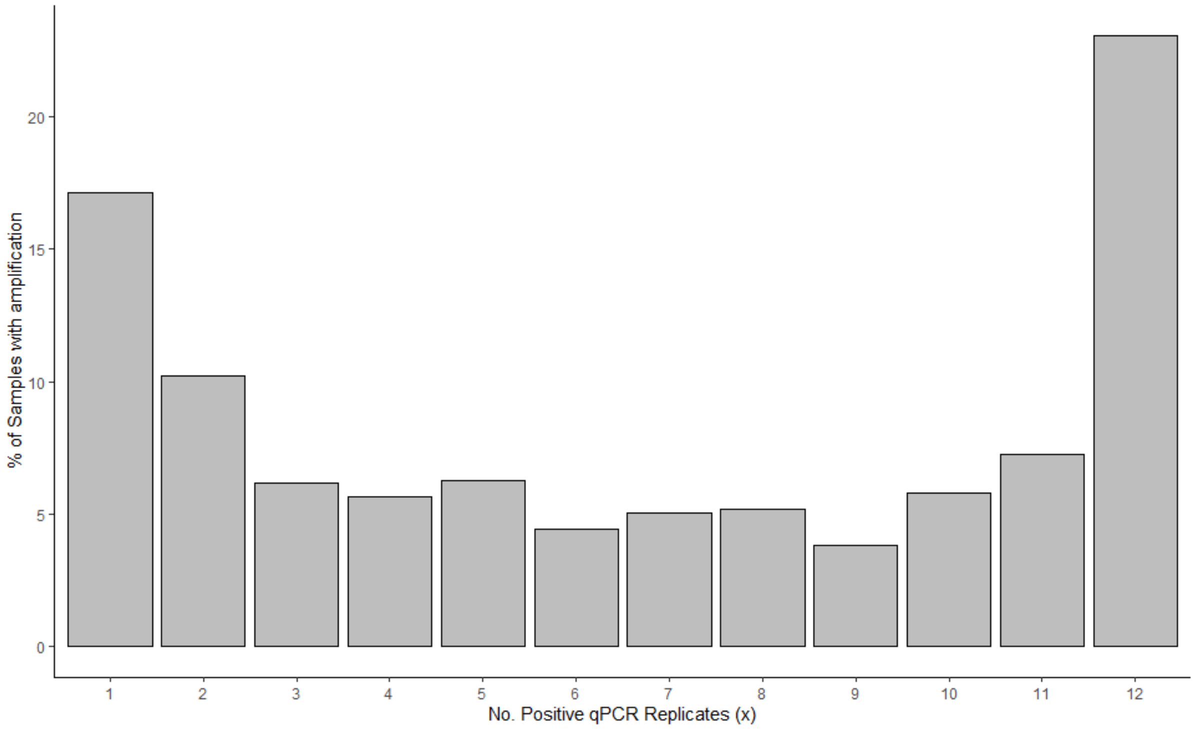


Figure S2 - Percentage of samples with x number amplifying qPCR replicates, excluding samples which showed no amplification.

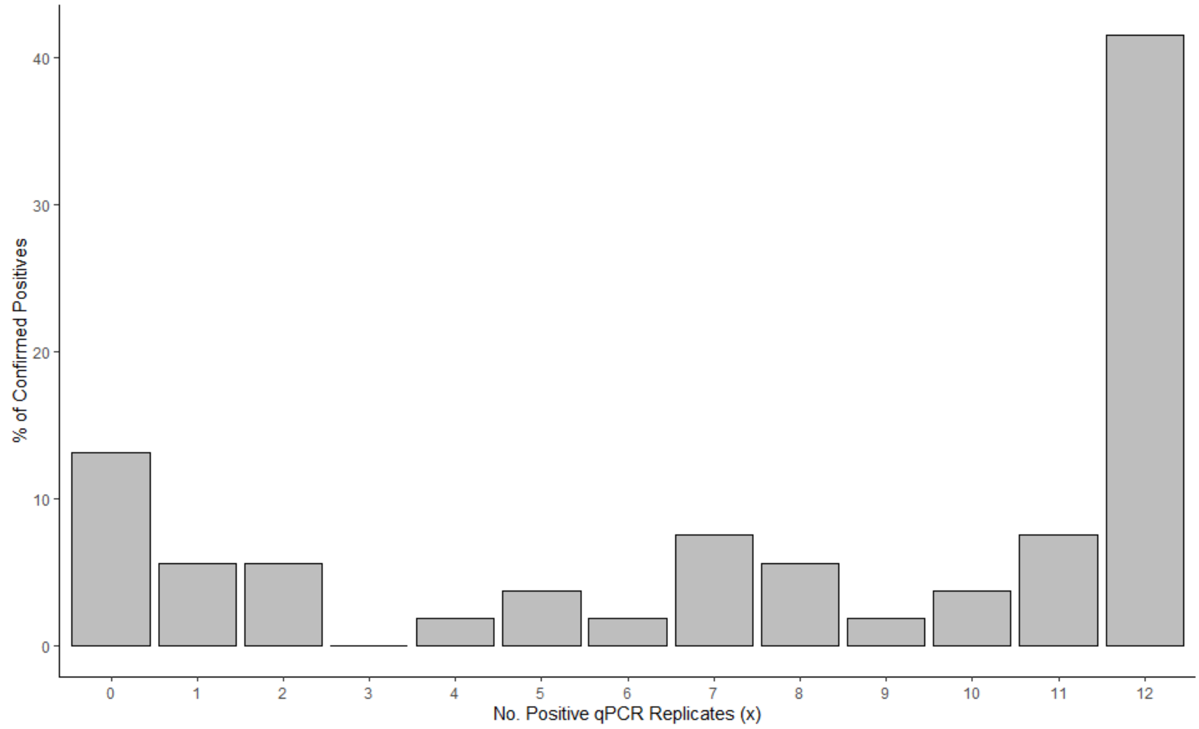


Figure S3 – The percentage of samples with confirmed presence, compared with the number of positive qPCR replicates for those sites.

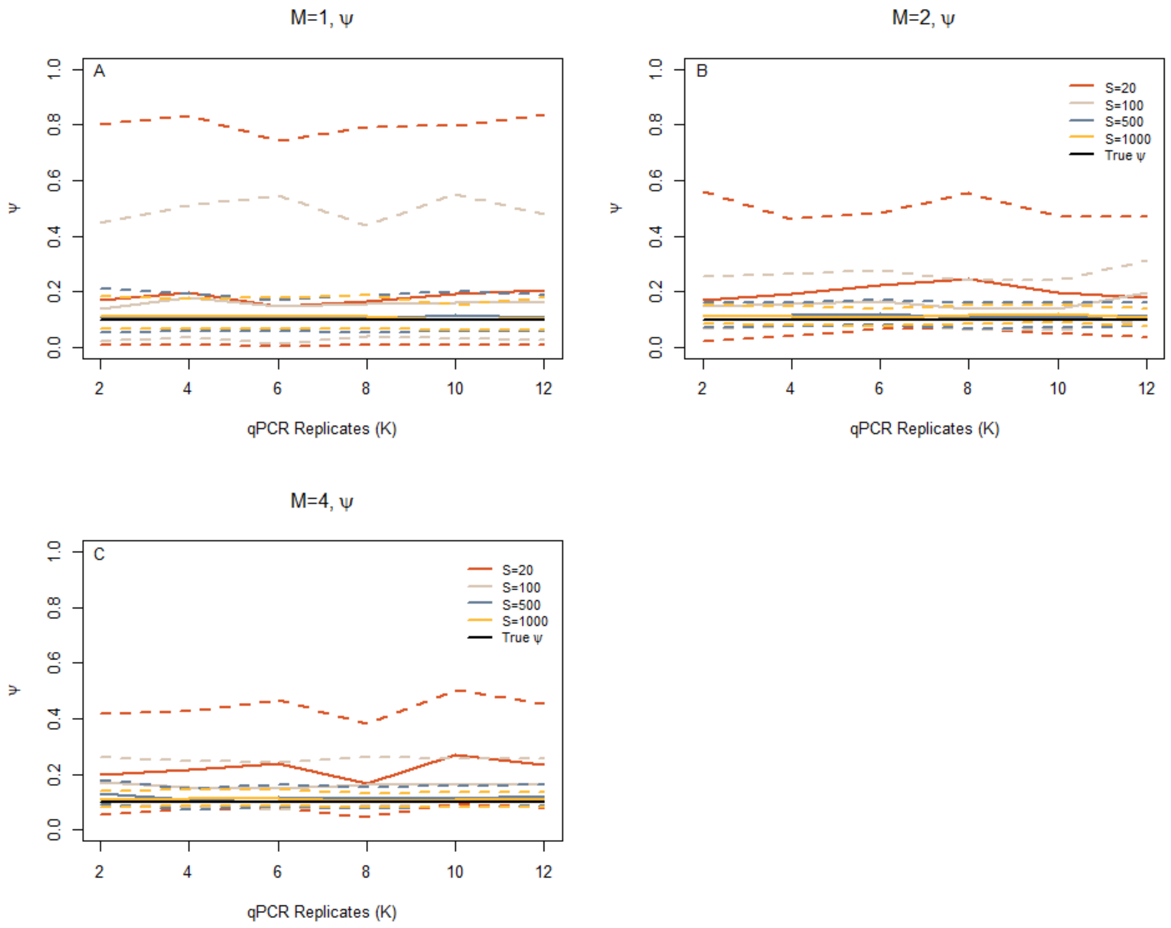


Figure S4 – Posterior mean ψ estimate (solid line) and 95% credible intervals (dashed lines) for each combination S (S=20, orange; S=100, grey; S=500, blue; S=1000, yellow), K (x-axis) and M (M=1, plot A; M=2, plot B; and M=4, plot C). The value used to simulate the data in each case is equal to 0.1.

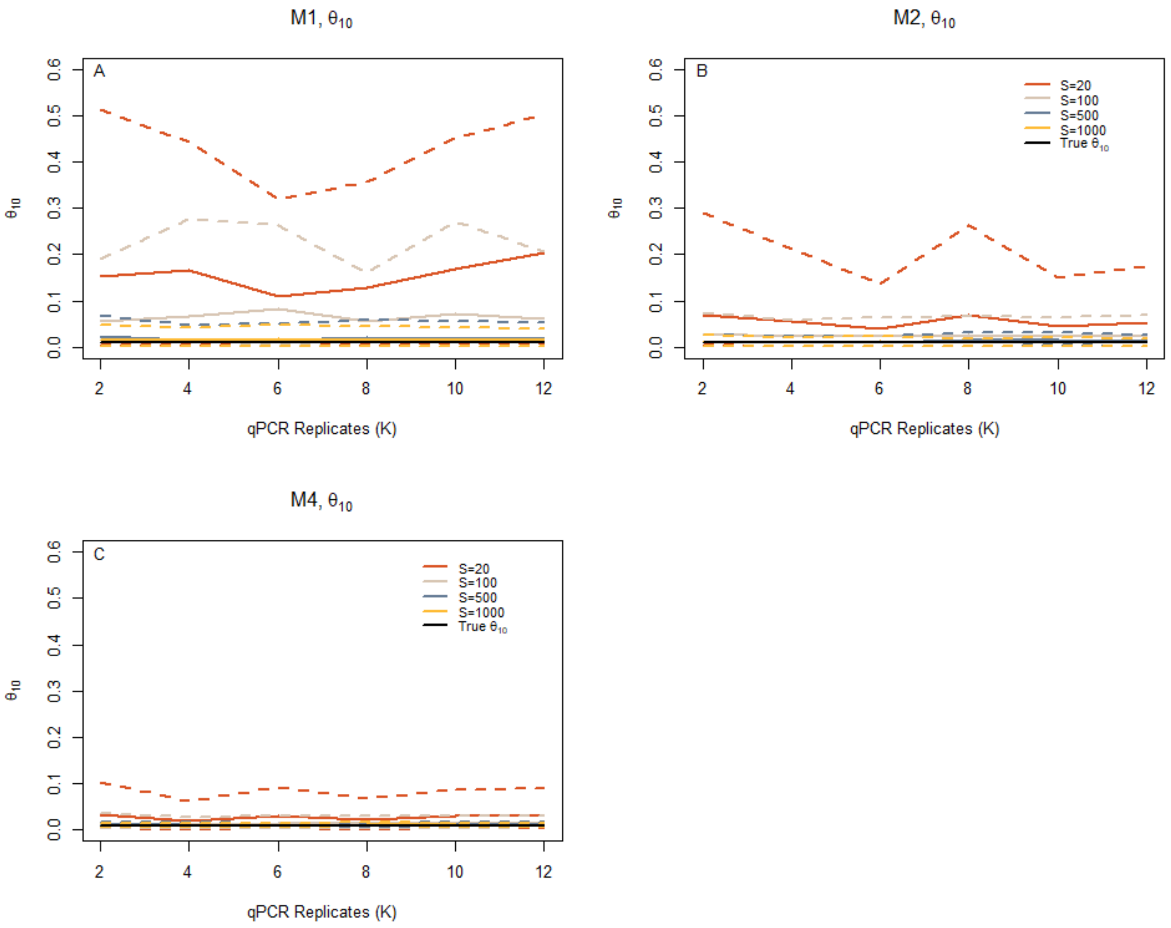


Figure S5 – Posterior mean $\theta_{10}$ estimate (solid line) and 95% credible intervals (dashed lines) for each combination S (S=20, orange; S=100, grey; S=500, blue; S=1000, yellow), K (x-axis) and M (M=1, plot A; M=2, plot B; and M=4, plot C). The value used to simulate the data in each case is equal to 0.01.

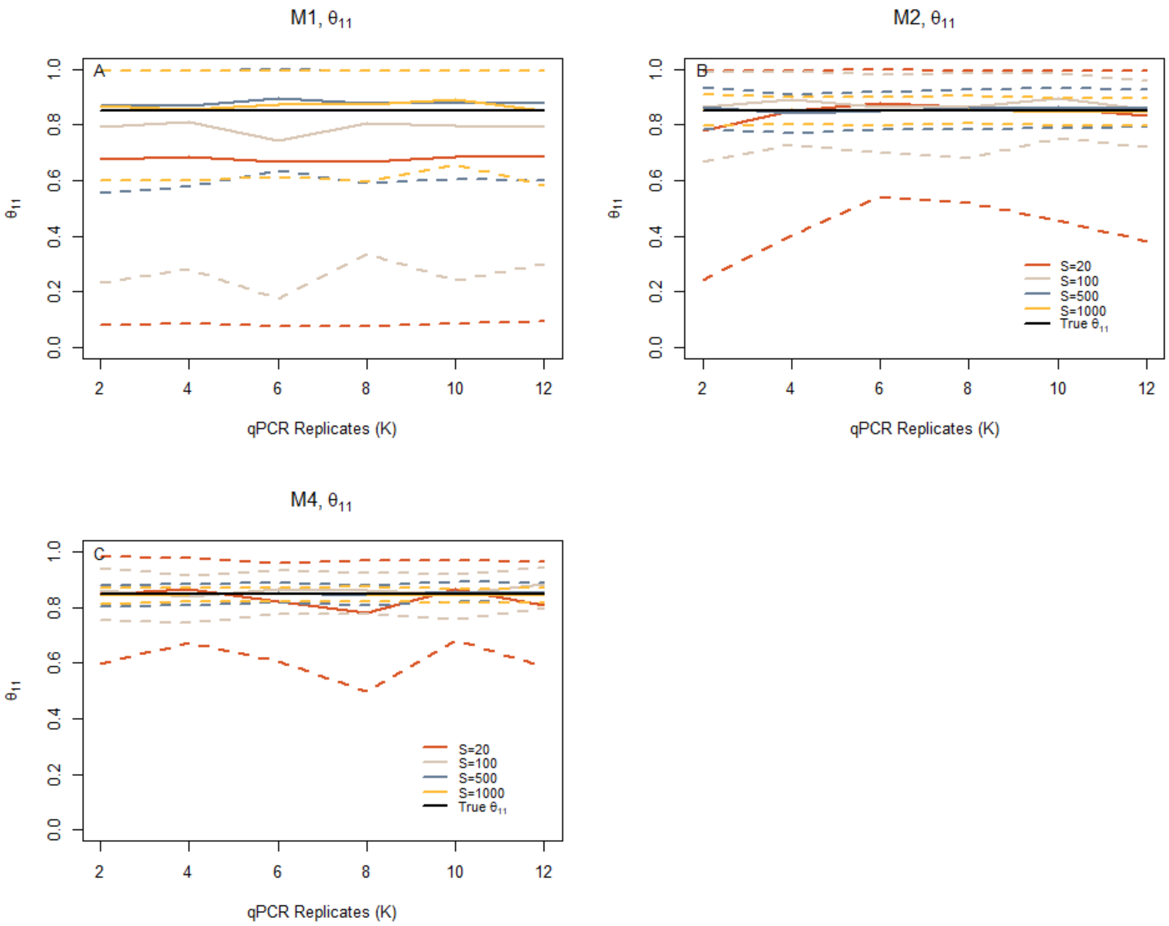


Figure S6 – Posterior mean $\theta_{11}$ estimate (solid line) and 95% credible intervals (dashed lines) for each combination S (S=20, orange; S=100, grey; S=500, blue; S=1000, yellow), K (x-axis) and M (M=1, plot A; M=2, plot B; and M=4, plot C). The value used to simulate the data in each case is equal to 0.85.

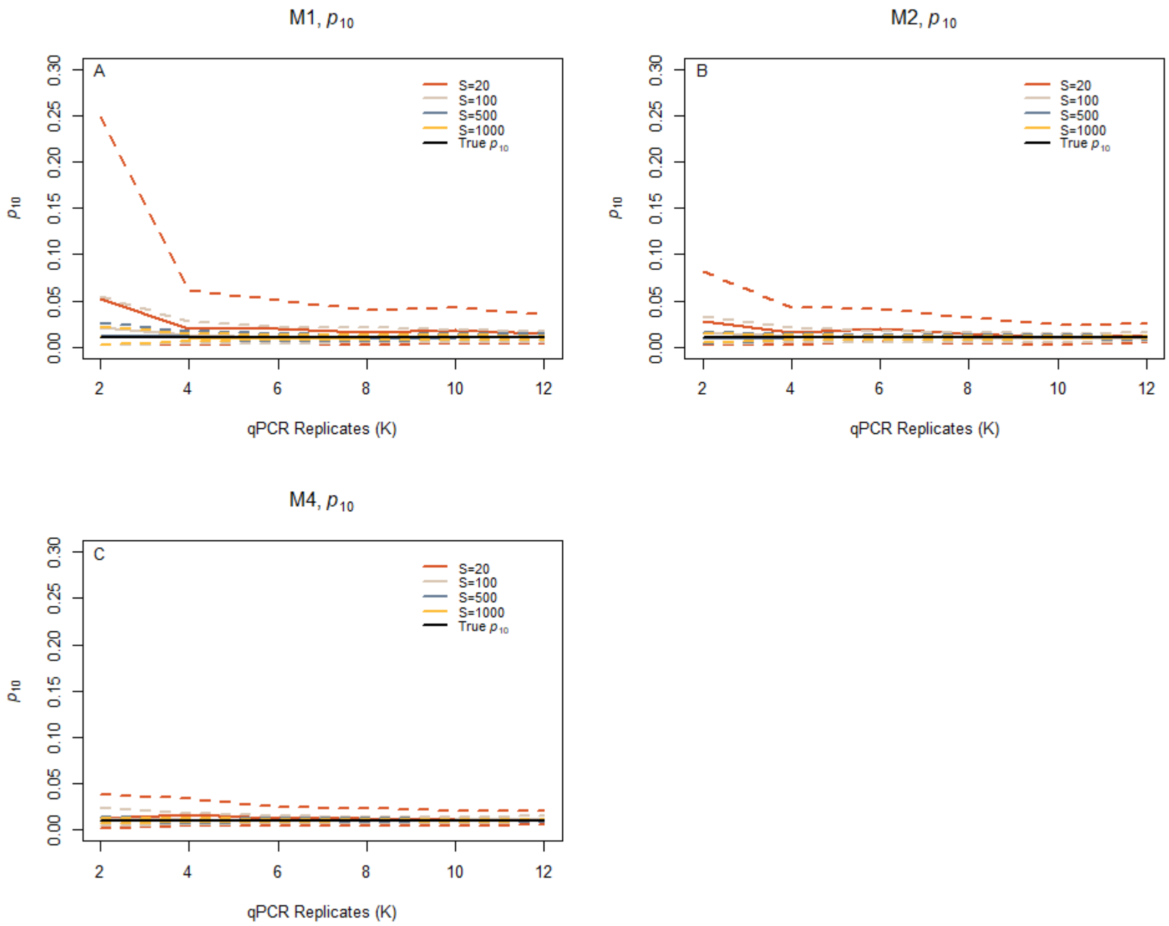


Figure S7 – Posterior mean $p_{10}$ estimate (solid line) and 95% credible intervals (dashed lines) for each combination S (S=20, orange; S=100, grey; S=500, blue; S=1000, yellow), K (x-axis) and M (M=1, plot A; M=2, plot B; and M=4, plot C). The value used to simulate the data in each case is equal to 0.01.

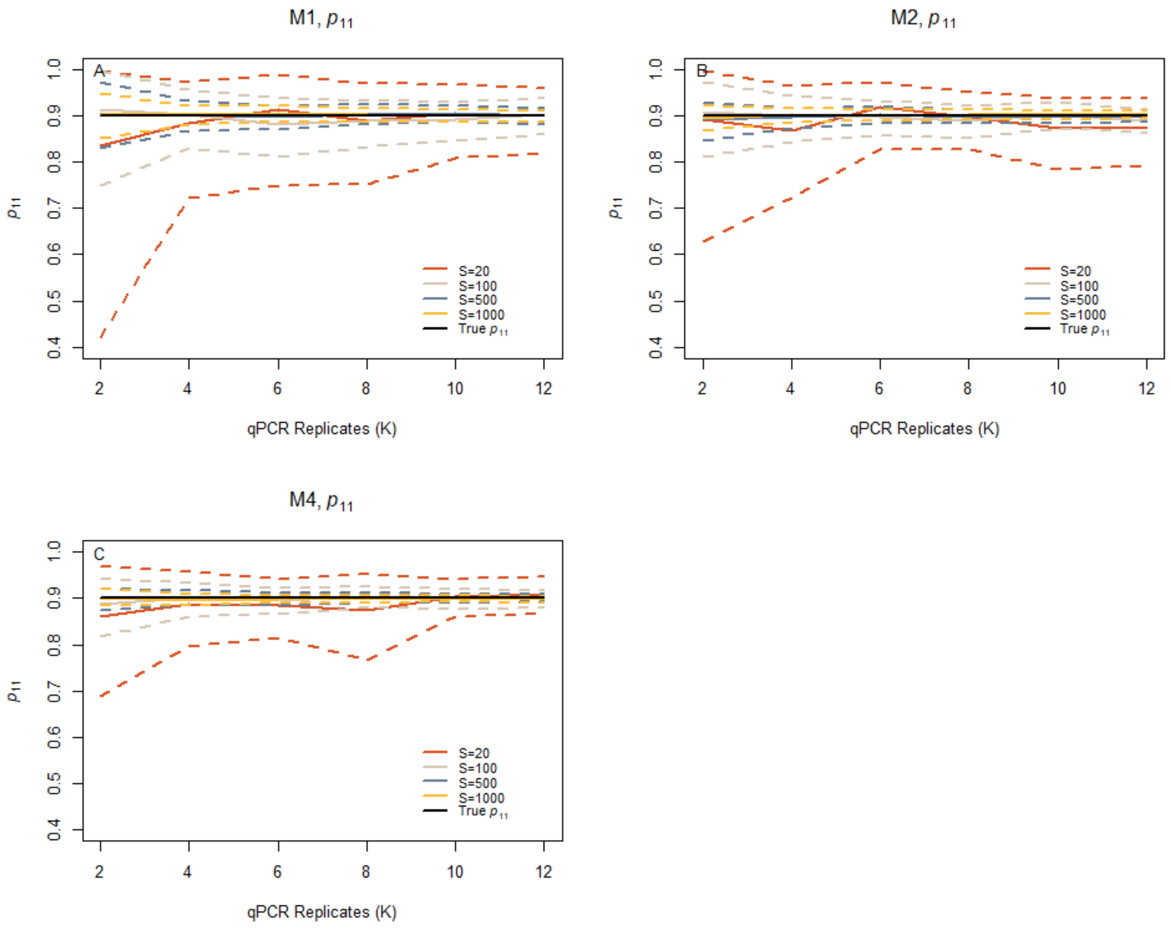


Figure S8 – Posterior mean $p_{11}$ estimate (solid line) and 95% credible intervals (dashed lines) for each combination S (S=20, orange; S=100, grey; S=500, blue; S=1000, yellow), K (x-axis) and M (M=1, plot A; M=2, plot B; and M=4, plot C). The value used to simulate the data in each case is equal to 0.9.
